# Mechanistic Fingerprints from Chloride to Iodide: Halide vs. Ammonia Release in Platinum Anticancer Complexes

**DOI:** 10.3390/ijms262412138

**Published:** 2025-12-17

**Authors:** Lorenzo Chiaverini, Luca Famlonga, Davide Piroddu, Matteo Pacini, Riccardo Di Leo, Emma Baglini, Damiano Cirri, Tiziano Marzo, Diego La Mendola, Alessandro Pratesi, Paola Ferrari, Andrea Nicolini, Alessandro Zucchi, Alessandro Marrone, Iogann Tolbatov

**Affiliations:** 1 Department of Pharmacy, University of Pisa, Via Bonanno Pisano 6, 56126 Pisa, Italy; lorenzo.chiaverini@phd.unipi.it (L.C.); luca.famlonga@phd.unipi.it (L.F.); diego.lamendola@unipi.it (D.L.M.); 2 Department of Chemical, Physical, Mathematical and Natural Sciences, University of Sassari, 07100 Sassari, Italy; d.piroddu@studenti.uniss.it (D.P.); tolbatov.i@gmail.com (I.T.); 3 Department of Translational Research and New Technologies in Medicine and Surgery, University of Pisa, Via Savi 10, 56126 Pisa, Italy; m.pacini31@studenti.unipi.it (M.P.); andrea.nicolini@med.unipi.it (A.N.); alessandro.zucchi@unipi.it (A.Z.); 4 National Council of Research (CNR), Institute of Clinical Physiology, 56124 Pisa, Italy; riccardo.dileo@cnr.it (R.D.L.); emma.baglini@cnr.it (E.B.); 5 Department of Chemistry and Industrial Chemistry (DCCI), University of Pisa, Via G. Moruzzi, 13, 56124 Pisa, Italy; alessandro.pratesi@unipi.it; 6 Department of Oncology, Pisa University Hospital, Via Roma 57, 56126 Pisa, Italy; p.ferrari@ao-pisa.toscana.it; 7 Department of Pharmacy, University “G d’Annunzio” Chieti-Pescara, Via dei Vestini 31, 66100 Chieti, Italy; amarrone@unich.it

**Keywords:** platinum complexes, cancer, cisplatin analog, NMR, DFT

## Abstract

Platinum-based drugs play a pivotal role in contemporary cancer treatment, but their therapeutic utility is often limited by acquired resistance. The diiodido analog, cis-[PtI_2_(NH_3_)_2_] is a promising derivative that has demonstrated the ability to overcome cisplatin resistance in vitro. To establish the molecular basis for this superior activity, we integrated experimental (NMR) spectroscopy with computational density functional theory (DFT) methods to precisely and comparatively understand the drug activation mechanisms. Comparative ^14^N NMR experiments elucidated the initial ligand substitution step, confirming halide displacement and a markedly higher tendency for ammonia release from cis-[PtI_2_(NH_3_)_2_], particularly when reacting with sulfur-containing amino acids. Complementary DFT calculations determined the substitution energy values, revealing that the superior leaving-group ability of iodide results in a thermodynamically more favorable activation. Conceptual DFT parameters (softness, hardness, and Fukui indices) further demonstrated that initial substitution induces a strong trans effect, leading to the electronic sensitization of the remaining iodide ligand. This strong agreement between computational predictions and experimental data establishes a coherent molecular activation mechanism for cis-[PtI_2_(NH_3_)_2_], demonstrating that iodide substitution promotes both thermodynamic and electronic activation of the platinum center, which is the key to its distinct pharmacological profile and ability to circumvent resistance.

## 1. Introduction

Platinum-based drugs represent one of the cornerstones of contemporary anticancer chemotherapy, with cisplatin being the paradigm and still one of the most widely used agents in clinical practice [1,2,3]. Despite its remarkable efficacy, the therapeutic utility of cisplatin is limited by intrinsic and acquired resistance, as well as by severe dose-dependent side effects [4,5,6]. While effective against various cancers, cisplatin is limited by severe adverse effects [7,8] and the widespread problem of intrinsic and acquired resistance [9]. To overcome these challenges, the search for better and more effective alternatives is essential, motivating research into new Pt complexes and other metallodrugs [10,11,12]. Also, starting from classical platinum drugs, the exploitation of different Pt oxidation states (e.g., Pt(IV)), is a reliable option because of the possibility of coupling additional ligands at the axial positions owing to the octahedral geometry. In this configuration, axial ligands are typically released under specific conditions (that are finely tunable) such as the reducing cellular environment [13,14]. In this context, provided the axial ligands are biologically active molecules, it is possible to exploit their delivery in combination of a Pt-based anticancer drug that undergoes reduction to cisplatin-like Pt(II) entity [15,16,17]. Additionally, several different transition metals are well exploitable for developing new and more efficient treatments [12,18,19,20]. For this reason, the development and mechanistic investigation of cisplatin analogs remain a highly active area of research, aiming both at improving pharmacological performance and at disclosing the molecular principles underlying platinum–biomolecule interactions [21,22,23].

**Figure 1:**
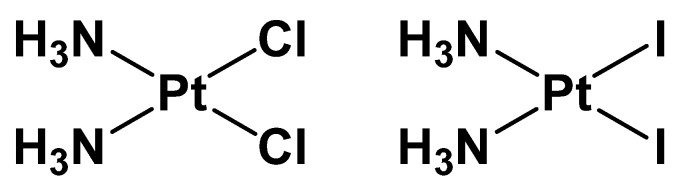
Chemical structure of cisplatin (**left**) and its iodide analog (**right**).

In this context, the diiodido analog of cisplatin, cis-[PtI_2_(NH_3_)_2_] (hereafter cisPtI_2_, Figure 1), has long been neglected, mainly due to early and largely unsubstantiated claims of poor pharmacological activity [24]. Our recent systematic studies, however, have contributed to reappraising the chemical and biological profile of cisPtI_2_. In particular, we have shown that this compound displays cytotoxicity comparable to cisplatin against several tumor cell lines and, importantly, is able to overcome platinum resistance in vitro [25]. Furthermore, while the DNA platination pattern of cisPtI_2_ closely mirrors that of cisplatin, subtle yet significant differences in its solution behavior and reactivity appear to be responsible for its distinct biological effects, notably its ability to overcome platinum resistance [25,26].

Previous studies highlighted how cisPtI_2_ differs from cisplatin in its interactions with proteins and model amino acid sidechains. Comparative experiments with lysozyme and cytochrome c revealed a divergent reactivity pattern, which was further rationalized by density functional theory (DFT) calculations on sulfur- and nitrogen-containing ligands. These studies demonstrated that while both cisplatin and cisPtI_2_ readily undergo halide substitution, the subsequent steps, including possible ammonia release, are markedly influenced by the nature of the incoming ligand [27]. Such findings pointed to the protein environment as a crucial determinant of the peculiar reactivity of cisPtI_2_ and of the nature of its adducts.

Building on these foundations, the present study aims to provide a deeper mechanistic understanding of the activation processes of cisplatin and cisPtI_2_ by combining complementary experimental and computational approaches. In particular, we employed ^14^N nuclear magnetic resonance (NMR) spectroscopy to monitor the earliest substitution steps, focusing on halide displacement and the potential release of coordinated ammonia. Complementary to this experimental work, DFT was used to calculate the Gibbs free energy of substitution—which assesses the thermodynamic stability of the Pt-biomolecule conjugates—along with conceptual DFT descriptors such as softness, hardness, and Fukui indices [28]. These calculations, performed using small-molecule models, correlate theoretical reactivity parameters with the experimentally observed ligand exchange behavior.

The integration of NMR spectroscopy with computational chemistry offers a coherent and complementary perspective on the ligand substitution processes of these platinum complexes. By elucidating the molecular basis of halide and ammonia release, this work contributes to a more precise definition of the activation pathways of cisplatin and cisPtI_2_. Ultimately, such knowledge is essential for rationalizing the distinct pharmacological properties of these complexes and for guiding the design of new platinum-based drugs with improved efficacy and resistance profiles.

## 2. Results and Discussion

### 2.1. Cisplatin vs. cisPtI_2_: NMR Investigation of Ligand Exchange with Histidine, Cysteine, and Methionine

The ligand substitution reactions of cisplatin and cisPtI_2_ with three representative amino acids (histidine, cysteine, and methionine) were investigated using NMR spectroscopy. Due to technical constraints and the risk of salt precipitation, the study was limited to the first substitution step, which is nevertheless the most relevant for understanding the distinct reactivity of these Pt complexes with small proteins, as previously reported [29,30,31]. All experiments were conducted in a 50% DMF/water mixture to ensure the solubility of all reagents throughout the reaction. The coordination behavior of the ammonia ligand, including its potential release from the platinum center, was monitored for both complexes by ^14^N NMR spectroscopy. ^14^N is a magnetically active nucleus (spin = 1) with a quadrupolar moment. Although this feature is often considered disadvantageous, we selected ^14^N because its faster magnetization recovery (T_1_ of ^14^N in ammonia is ~25 times shorter than that of ^15^N) enables the acquisition of thousands of scans within a relatively short time, yielding spectra with an excellent signal-to-noise ratio. However, the linewidth of ^14^N signals increases proportionally with lattice anisotropy around the observed nucleus, restricting the applicability of this technique mainly to highly symmetrical molecules. In the case of the two Pt complexes, the ^14^N NMR spectra typically displays a broad resonance attributable to platinum-coordinated ammonia. Upon the release of ammonia from the metal center, a sharp signal corresponding to the NH_4_^+^ cation emerges at −23 ppm, allowing a quantitative assessment of ammonia release by NMR spectroscopy (Table 1).

As shown in Figures S1–S4, it is evident that the two complexes upon dissolution in the reference medium are stable over time and that no ammonia release occurs. The stability of the ^14^N NMR signal also suggests that no halide release occurs under the applied experimental conditions. This observation contrasts with our previous findings on halide release from the two Pt compounds under physiological-like conditions [25]. However, in our case, the different behavior is attributable to the relatively high percentage of DMF, which disfavors halide displacement, and to the relatively large concentration of the two complexes.

**Figure 2:**
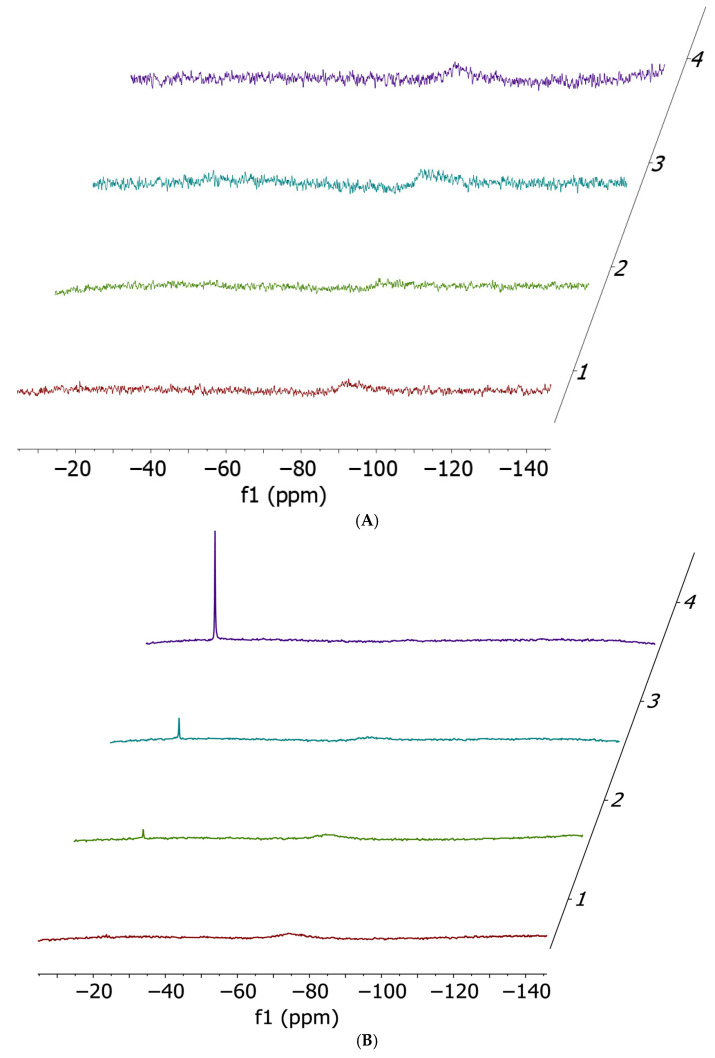

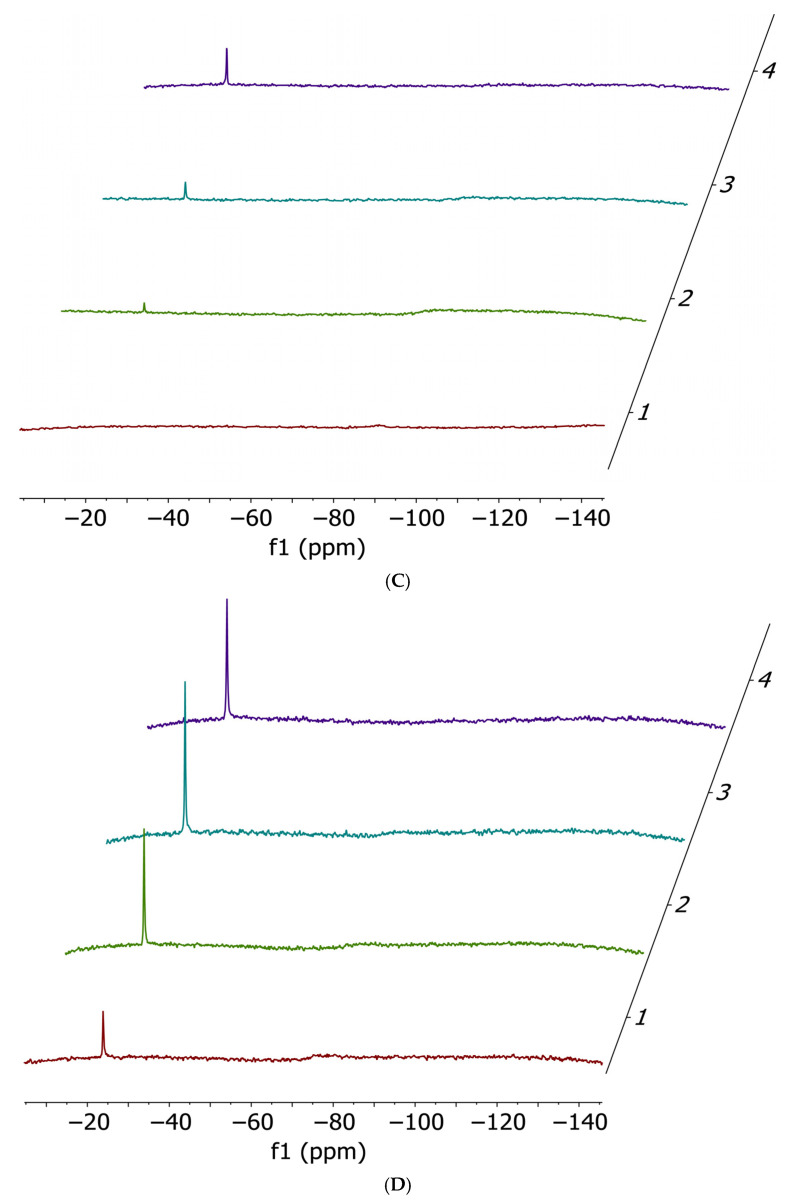

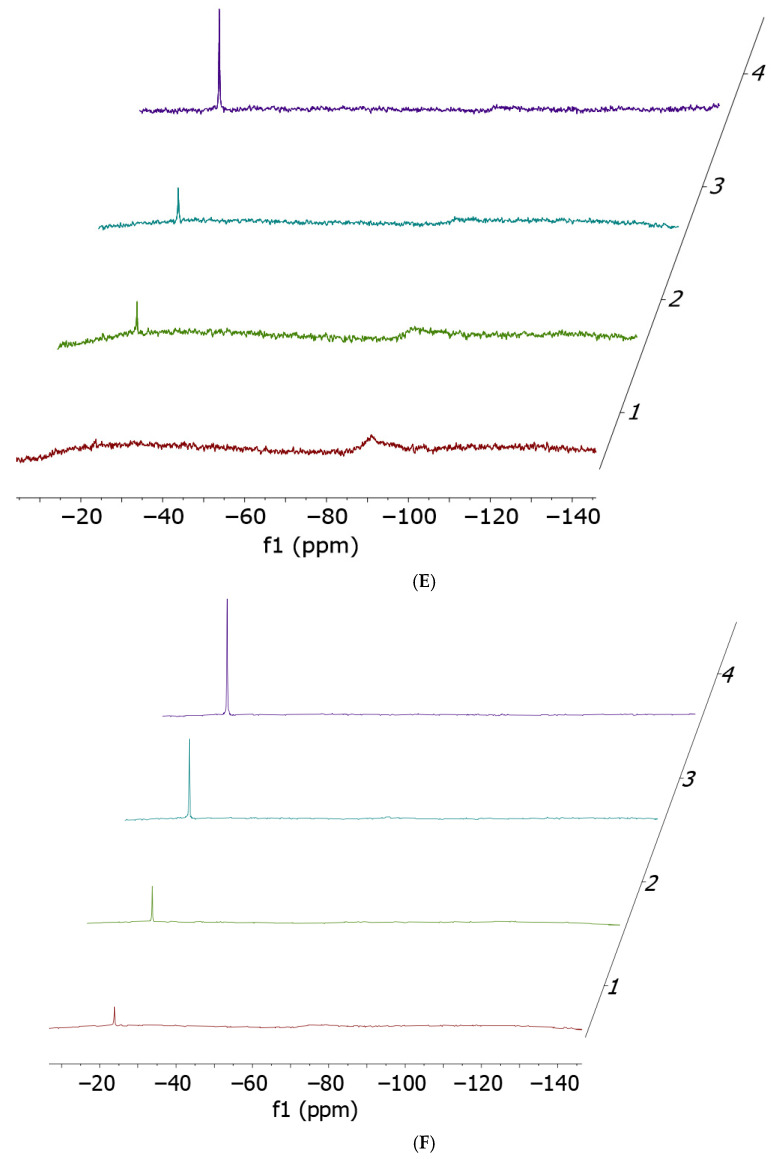
Ammonia release ^14^N NMR spectra performed at t0 (red), 2 h (green), 5 h (cyan) and 24 h (violet). Cis-[PtCl_2_(NH_3_)_2_] 17 mM (**A**) and cisPtI_2_ 34 mM (**B**) incubated with 1 equivalent of histidine; cis-[PtCl_2_(NH_3_)_2_] 17 mM (**C**) and cisPtI_2_ 34 mM (**D**) incubated with 1 equivalent of methionine; cis-[PtCl_2_(NH_3_)_2_] 17 mM (**E**) and cisPtI_2_ 34 mM (**F**) incubated with 1 equivalent of cysteine. All spectra have been acquired in a solvent mixture of 250 µL of DMF-d_7_ and 250 µL of Buffer Acetate 100 mM pH 6.9.

Upon addition of 1 equivalent of each amino acid to both cisplatin or cis-[PtI_2_(NH_3_)_2_], the ^14^N NMR spectra depicted in Figure 2 are obtained.

^14^N NMR experiments confirmed the occurrence of ammonia release (−23 ppm) in almost all cases, with the only exception being the incubation of cisplatin with histidine. Quantitatively, the preferential ammonia release from cisPtI_2_ (−75 ppm, broad) with respect to cisplatin (−91 ppm, broad) is clearly demonstrated by the significantly higher NH_4_^+^ peak heights.

### 2.2. Computational Assessment

The present study employs a purely thermodynamic approach, calculating the Gibbs free energy of substitution (GFE) for the reaction in which reactants and products are infinitely separated. This methodology differs significantly from the pseudounimolecular approach used in our previous work on cisplatin and cisPtI_2_ [27], which focused exclusively on the reaction profile including reactant-adduct, transition-state, and product-adduct species. While the prior work provided insight into kinetic barriers, the current free energy values reflect the inherent thermodynamic driving force for the overall substitution process, providing important insight into the relative stability of the resulting Pt-biomolecule conjugates at equilibrium. To accurately reflect the experimental conditions (DMF/acetate buffer), all presented data in the main text (Table 2, Table 3, Table 4 and Table 5) and the discussion below were calculated using the implicit N,N-dimethylformamide (DMF) solvent model. Complete results calculated in water are provided in the Supporting Information (Tables S1 and S2).

A comprehensive understanding of the complex’s reactivity requires the integration of both kinetic (rate) and thermodynamic (stability) data, an approach critical for a complete mechanistic picture, as emphasized also in [32]. Indeed, the kinetic data from our previous study [12] strongly corroborates the thermodynamic trends presented here. Specifically, the superior leaving-group ability of I^−^, which leads to favorable GFE values, is quantitatively confirmed by significantly lower activation free energies for halide substitution in cisPtI_2_ compared to cisplatin. Furthermore, the enhanced propensity for NH3 labilization, which is the defining feature of cisPtI_2_’s unique pharmacological profile, is confirmed kinetically by significantly reduced kinetic barriers for ammine substitution that are 4–6 kcal/mol lower than those for cisplatin. This significant difference in kinetic barriers is a direct consequence of the strong trans-effect of iodide and provides the mechanistic basis for the compound’s rapid activation, a phenomenon consistent with the findings of Sicilia et al. [32] that link the increased soft character, leaving, and bridging abilities of iodide to the unique cytotoxic profile.

A systematic comparison of the substitution reaction GFE energies calculated in DMF (Table 2 and Table 3) immediately highlights a substantial thermodynamic advantage for iodoplatin over cisplatin. For all three neutral, biologically relevant nucleophile models—thiol (CH_3_SH), dimethyl sulfur (CH_3_SCH_3_), and imidazole (imi)—substitution is thermodynamically more favorable for the iodo-complexes. This thermodynamic advantage stems directly from the intrinsically superior leaving group ability of the iodide (I^−^) ligand compared to chloride (Cl^−^). This fundamental difference shifts the substitution reaction to become significantly less endothermic or more exothermic in the iodoplatin series. For instance, substitution by the neutral thiol model is highly endothermic for cisplatin (GFE = +7.5 kcal/mol in DMF), yet for iodoplatin, the reaction is only mildly endothermic (GFE = +2.1 kcal/mol), representing an approximate 5.4 kcal/mol thermodynamic driving force differential. Crucially, the overall order of substitution favorability remains consistent for both parent compounds: imidazole ≫ dimethyl sulfur > neutral thiol. The most thermodynamically favorable substitution observed among the neutral nucleophiles is the replacement of iodide by imidazole (GFE = −7.6 kcal/mol), reflecting the strong binding ability of the imidazole nitrogen to Pt(II). This robust thermodynamic preference for N-donor sites supports and quantitatively extends observations learned from the kinetic analysis in the iodoplatin study [27].

A direct comparison between the experimental ^14^N NMR findings (Table 1) and the GFE data (Table 3) reveals an apparent kinetic/thermodynamic discrepancy regarding cysteine reactivity with the cisPtI_2_ complex. Specifically, the reaction with cysteine is the most kinetically facile (100% NH_3_ release), yet the computational model utilizing the neutral thiol suggests the product is thermodynamically disfavored (GFE = +2.1 kcal/mol). This discrepancy is fully resolved by the inclusion of the thiolate anion (CH3S^−^), which models the deprotonated cysteine.

The substitution of iodide by the thiolate anion is highly exothermic (GFE = −24.4 kcal/mol in DMF). This strong thermodynamic favorability demonstrates that the true product observed at equilibrium is the highly stable Pt-thiolate conjugate. The mechanistic path thus involves a sequence where the initial binding step (substitution by either R-SH or the available R-S^−^) is followed by a rapid deprotonation of the coordinated thiol (Pt-SH → Pt-S^−^) due to the significant increase in thiol acidity upon coordination to Pt(II). The resulting Pt-thiolate bond then exerts a maximal trans-effect on the NH_3_ ligand, driving the rapid, subsequent release observed in the ^14^N NMR experiments. Therefore, the fast ammonia release is a kinetic consequence of forming the Pt-thiolate, which is the overwhelmingly favorable thermodynamic product.

The computational trends observed in DMF are robustly maintained in the aqueous solvent model, suggesting that the intrinsic reactivity differences are retained in the physiological environment (see SI and compare with Tables S1 and S2). The discrepancy between the kinetic observation (^14^N NMR monitoring subsequent NH_3_ release, driven by the strong trans-effect) and the initial substitution equilibrium (GFE of the neutral thiol adduct) is reconciled by the overwhelming stability of the final Pt-thiolate product. The experimental observation of maximal NH_3_ release from the cysteine adduct suggests that once the initial Pt-S bond forms, the resulting intermediate complex is significantly more prone to fast, subsequent NH_3_ release than the corresponding methionine or histidine adducts, a phenomenon that is kinetically favored despite the less favorable initial substitution equilibrium.

Iodoplatin exhibits a significantly lower chemical hardness (η = 0.147 in DMF) and a commensurately higher global softness (S = 3.391 in DMF) compared to cisplatin (η = 0.169 in DMF, S = 2.957 in DMF). This electronic profile indicates that iodoplatin is an intrinsically softer molecule, which aligns with the principle of hard-soft acid-base (HSAB) theory and suggests a strong preference for interaction with soft, polarizable nucleophiles, such as the sulfur donors (CH_3_SH, CH_3_SCH_3_). This intrinsic electronic softness provides a direct, quantitative rationale for the experimentally observed enhanced reactivity: the HSAB alignment with soft sulfur donors (cysteine and methionine) predicted by this lower hardness is confirmed by the significantly greater NH_3_ release seen in the ^14^N NMR studies (Table 1). Furthermore, the significantly greater $f^{-}$ index value on the iodide atom compared to chloride directly correlates with its superior leaving-group ability, providing the electronic basis for the faster overall ligand displacement seen in the experimental analysis. The increased softness quantitatively supports the kinetic lability implied by the GFE data, confirming that iodoplatin is electronically primed for substitution. Furthermore, the chemical potential (μ) shifts to more negative values upon substitution by sulfur ligands for both cisplatin and iodoplatin, suggesting the resulting cationic products are stronger electron acceptors. The electrophilicity index (ω), a measure of the complex’s capacity to acquire electrons, generally increases upon substitution, with the substituted iodoplatin complexes, [Pt(NH_3_)_2_I(L)]^+^, showing the highest values (up to ω = 0.087 for the CH_3_SH adduct in DMF). This heightened electrophilicity indicates that the positively charged substitution products are not inert, but rather highly reactive species with an increased propensity for subsequent binding events, such as coordination to a second nucleophile or cross-linking DNA.

The demonstrated intrinsic electronic softness of cisPtI_2_ (η) and the superior thermodynamic favorability of substitution by sulfur donors (Table 3) have critical pharmacokinetic implications. Glutathione (GSH), an abundant tripeptide containing a highly reactive soft thiol group, is the primary biological detoxification agent for platinum drugs [33]. Based on the HSAB principle, which predicts soft acids (like cisPtI_2_) will react faster with soft bases (like the GSH thiolate), the increased lability of cisPtI_2_ suggests it is intrinsically more susceptible to rapid inactivation by GSH than cisplatin. While this increased reactivity poses a challenge for maintaining therapeutic concentrations in vivo, this very lability is what drives the compound’s enhanced activation kinetics, especially in the low-GSH tumor microenvironment, where its therapeutic benefit is realized through rapid DNA binding and its ability to circumvent resistance mechanisms.

To understand the specific electronic reorganization driving the substitution, we analyzed the local reactivity using Fukui indices ($f^{+}$ and $f^{-}$), which quantify the change in electron density at specific atomic sites upon gaining or losing an electron (Table 4 and Table 5, calculated in DMF; Tables S3 and S4 in water). The $f^{+}$ index describes local electrophilicity (susceptibility to nucleophilic attack at the Pt center), and $f^{-}$ describes local nucleophilicity (electron-donating ability, relevant for the halide leaving groups). The calculated Fukui indices confirm the electronic basis for the thermodynamic preference of ligand substitution in cisplatin and iodoplatin.

Analysis of the leaving group character, represented by $f^{-}$, reveals that for the parent compounds [Pt(NH_3_)_2_X_2_], the negative value of $f^{-}$ is significantly greater on the iodide atoms (X = I, $f^{-}$ = −0.287) than on the chloride atoms (X = Cl, $f^{-}$ = −0.104). This 176% larger magnitude of $f^{-}$ on the iodide atom signifies a correspondingly greater capacity to donate electron density (be lost as I^−^), confirming its superior leaving group ability in the initial substitution step.

In terms of the central Pt atom’s reactivity, the Pt center in cisplatin exhibits a more negative $f^{+}$ value (−0.410) than that in iodoplatin (−0.300), suggesting the Pt atom in cisplatin is, unexpectedly, a slightly stronger electron acceptor in its initial state. However, the overall reaction outcome is decisively dominated by the facile release of the iodide ligand, as confirmed by the favorable GFE data.

Upon monosubstitution to form [Pt(NH_3_)_2_X(L)]^+^, a notable electronic and mechanistic consequence is observed: the $f^{-}$ value on the remaining halide (X) is significantly enhanced in magnitude, particularly for the remaining iodide atom (X = I) when a neutral sulfur donor is trans-positioned. The $f^{-}$ on I drops to values as low as −0.637 when the nucleophile is CH_3_SH. This 122% increase in the magnitude of the $f^{-}$ index on the remaining iodide signifies a powerful electronic activation, essentially demonstrating an augmented trans-effect from the newly bound neutral sulfur ligand. In contrast, while the Pt-thiolate complex is the thermodynamic product (GFE = −24.4 kcal/mol), the $f^{-}$ value on the remaining iodide is significantly lower (−0.089), suggesting that this highly stable product is less prone to subsequent I^−^ release than the neutral S-donor adducts. However, the thiolate’s strong trans-effect is electronically confirmed by the significant positive $f^{-}$ observed on one of the coordinated NH_3_ ligands (up to +0.022 in [Pt(NH_3_)_2_I(CH_3_S^−^)]), which is consistent with the rapid NH_3_ labilization observed experimentally. This potent local electronic effect provides a robust molecular explanation for the distinct reactivity pattern of iodoplatin with proteins observed in initial experimental studies (as discussed in [27]), where the substitution leads to a product that is not just thermodynamically stable but is also electronically primed for subsequent, rapid binding events, a characteristic essential for achieving potent biological activity.

Overall, by integrating an unconventional NMR-based approach with conceptual DFT descriptors, we sought to directly probe the reactivity of the complexes and to reinforce previous evidence indicating that a significant difference indeed exists in the so-called in the interaction with amino acids and proteins, between cisplatin and its iodide analog. The claim that differences in the binding of biological substrates -and specifically proteins and amino acids- originate from previous publications. In 2015 we reported that very subtle differences exist in the metalation mode of DNA, based on the comparative analysis of ctDNA adducts formed by cisplatin and its iodide analog [25]. In contrast, remarkable differences occur in the protein metalation process, where the iodide analog preferentially releases the amine ligand instead of the halides. This represents a significant mechanistic divergence that may critically affect the pharmacological profile. Indeed, this behavior correlates with experimental evidence showing that the iodide analog can circumvent platinum resistance in colorectal cancer models. The distinct protein metalation pathway associated with the iodide analog was also structurally confirmed [34].

Taken together, these findings outline a highly promising biological behavior for cisPtI_2_ and strongly support its further evaluation in suitable preclinical settings. In addition, we believe that the data offer meaningful insights into the presumed mechanism of action of cisplatin and its derivatives. Considering that the DNA lesions induced by cisPtI_2_ are fewer in number and comparable in nature to those generated by cisplatin, the notion that its cytotoxic activity may involve pathways other than direct DNA damage gains further credibility. Within this perspective, the profoundly different pattern of protein metalation displayed by the iodide analog stands out as a plausible determinant of its capacity to bypass platinum resistance.

## 3. Materials and Methods

*Synthesis of compounds and ^14^N NMR experiments.* Cisplatin was purchased from Merck (Via Monte Rosa, 93 20149 Milano Italy) and used without further purification, cisPtI_2_ was synthesized as previously reported by some of us [25]. NMR spectra were recorded on a Bruker Avance III 400 spectrometer equipped with a Bruker Ultrashield 400 Plus (Viale V. Lancetti, 43, 20158 Milano, Italy) superconducting magnet (resonating frequencies: 400.13 and 28.89 MHz for ^1^H and ^14^N, respectively) and a 5 mm PABBO BB-1H/D Z-GRD Z108618/0049 probe (Viale V. Lancetti, 43, 20158 Milano, Italy). All experiments were run at room temperature (25 ± 2 °C) with a standard 1D sequence with inverse gated decoupling (zgig). All spectra were recorded as a mean of 2000 scans, with recycle delay of 2 s. All samples used in ^14^N NMR experiments were prepared in a solvent mixture of 250 µL of DMF-d_7_ and 250 µL of Buffer Acetate 100 mM pH 6.9. Quantitative determination of ammonia release at 24h was performed comparing absolute integrals of NH_4_^+^ signals with respect to a 100 mM NH_4_Br standard solution. The comparison was performed through nmrq function of Topspin 4.0.1 software.

*Computational details.* Our computations were based upon density functional theory (DFT), a proven methodology for accurately modeling the geometry and reaction characteristics of transition metal compounds [35,36,37], including those containing platinum [38,39]. All calculations were carried out employing the Gaussian 16 quantum chemistry package [40]. We utilized the range-corrected density functional ωB97X-D [41] throughout, selecting it for its known precision in determining both molecular structures and electronic energies [42]. Geometries of all molecules were optimized, and their initial electronic and solvation energies calculated, using the ωB97X-D functional paired with the def2SVP basis set [43,44] in DMF and in water. To account for the surrounding solvent environment, the polarizable continuum model (PCM) in its integral equation formalism variant (IEFPCM) [45] was applied. This specific model was chosen because it has recently demonstrated significantly reduced errors for the aqueous solvation free energies of both neutral and ionic species compared to alternative continuum models [46]. For the solvation energy of the chloride ion in water, the experimental value of −74.5 kcal/mol was employed [47]. This decision was made to account for the unique effects related to the protic nature of water that the implicit PCM approach may not fully assess, ensuring the most accurate representation of the chloride ion in an aqueous environment for the solvent model comparison. To ensure the consistency and validity of the comparison with the robust experimental data, it is noted that while our DFT calculations employed an implicit solvation model, the primary mechanistic findings—namely the superior I^−^ leaving group and the strong trans effect—are governed by intrinsic electronic factors and the large thermodynamic difference (~2–5 kcal/mol), making the overall reaction ordering and the validated experimental trends highly reliable and robust against minor variations in the mixed solvent environment [48]. Following geometry optimization, frequency calculations were performed to confirm the stability of the optimized structures and to obtain the necessary Zero-Point Energy (ZPE) and thermal corrections for calculating thermodynamic properties. The final, high-level single-point electronic and solvation energies were then computed on the optimized geometries using the ωB97X-D functional in conjunction with the larger def2TZVP basis set [43,44].

To comprehensively characterize the electronic reactivity of the compounds, we employed conceptual DFT (cDFT) [49,50]. The global reactivity parameters—specifically the chemical potential (μ), hardness (η), softness (S), and electrophilicity (ω)—were derived from the energies of the highest occupied molecular orbital (E_HOMO_) and the lowest unoccupied molecular orbital (E_LUMO_), as determined at the ωB97X-D/def2SVP level of theory in water. These parameters are reported in Hartree (Ha), with the exception of softness (S), which is in Ha^–1^. The parameters were calculated using the following established formulas: the chemical potential μ was approximated as $\frac{1}{2}\left(E_{HOMO}+E_{LUMO}\right)$; the hardness η was approximated as $\frac{1}{2}\left(E_{LUMO}-E_{HOMO}\right)$; the softness *S* was defined as $\frac{1}{2\eta }$; and the electrophilicity ω was calculated as $\frac{\mu^{2}}{2\eta }$.

The local reactivity at each atomic center was assessed by calculating the dimensionless Fukui indices (f) [51]. These indices were determined from the Mulliken charges (q) for a given atom k, which were calculated at the same ωB97X-D/def2SVP level of theory in water. The formulas used for the Fukui indices for nucleophilic ($f_{k}^{+}$) and electrophilic ($f_{k}^{-}$) attack were: $f_{k}^{+}=q_{k}\left(N+1\right)-q_{k}(N)$ and $f_{k}^{-}=q_{k}\left(N\right)-q_{k}(N-1)$, respectively, where N, N+1, and N−1 denote the number of electrons in the neutral, anionic, and cationic species.

## 4. Conclusions

The combined experimental and computational investigation presented here provides new mechanistic insights into the activation pathways of cisplatin and its iodide analog, cisPtI_2_. ^14^N NMR results revealed a markedly higher tendency of the iodide derivative to undergo ammonia release, particularly in the presence of sulfur-containing amino acids. Complementary DFT calculations demonstrated that the enhanced reactivity of cisPtI_2_ arises from its lower chemical hardness and the superior leaving-group ability of iodide, resulting in significantly more favorable substitution thermodynamics. Conceptual DFT descriptors and Fukui function analysis confirmed this, highlighting the intrinsic electronic softness of the iodinated species and the strong trans effect induced by the newly bound nucleophile on the remaining iodide ligand. Together, these findings outline a consistent picture in which iodide substitution promotes both thermodynamic and electronic activation of the platinum center, ultimately explaining the distinct reactivity and potent biological activity, including its ability to overcome cisplatin resistance, compared to cisplatin. The present results contribute to a deeper understanding of halide-dependent activation mechanisms and may guide the rational design of next-generation platinum-based anticancer agents with improved performance and resistance profiles.

## Figures and Tables

**Table 1:** Quantitative determination (%) of released ammonia at 24 h of incubation.

Cisplatin + His	Cisplatin + Met	Cisplatin + Cys	*cis*-[PtI_2_(NH_3_)_2_] + His	*cis*-[PtI_2_(NH_3_)_2_] + Met	*cis*-[PtI_2_(NH_3_)_2_] + Cys
No release	11%	14%	54%	41%	100%

**Table 2:** Gibbs free energies of substitution (GFE in kcal/mol) and conceptual DFT global descriptors (μ, η, S, ω) for cisplatin and monosubstituted analogs. In DMF.

Complex	GFE	μ	η	S	ω
[Pt(NH_3_)_2_Cl_2_]	0.0	−0.140	0.169	2.957	0.058
[Pt(NH_3_)_2_Cl(CH_3_SH)]	7.5	−0.163	0.168	2.968	0.079
[Pt(NH_3_)_2_Cl(CH_3_S^−^)]	−18.6	−0.113	0.154	3.249	0.041
[Pt(NH_3_)_2_Cl(CH_3_SCH_3_)]	2.7	−0.162	0.170	2.944	0.077
[Pt(NH_3_)_2_Cl(imi)]	−1.8	−0.146	0.177	2.821	0.060
[Pt(NH_3_)Cl_2_(CH_3_SH)]	4.9	−0.153	0.163	3.073	0.072
[Pt(NH_3_)Cl_2_(CH_3_S^−^)]	−15.7	−0.099	0.150	3.329	0.033
[Pt(NH_3_)Cl_2_(CH_3_SCH_3_)]	0.6	−0.153	0.164	3.056	0.071
[Pt(NH_3_)Cl_2_(imi)]	−1.7	−0.137	0.169	2.956	0.056

**Table 3:** Gibbs free energies of substitution (GFE in kcal/mol) and conceptual DFT global descriptors (*μ*, η, S, ω) for cisPtI_2_ and monosubstituted analogs.

Complex	GFE	*μ*	*η*	*S*	*ω*
[Pt(NH_3_)_2_I_2_]	0.0	−0.149	0.147	3.391	0.075
[Pt(NH_3_)_2_I(CH_3_SH)]	2.1	−0.163	0.152	3.285	0.087
[Pt(NH_3_)_2_I(CH_3_S^−^)]	−24.4	−0.122	0.147	3.408	0.051
[Pt(NH_3_)_2_I(CH_3_SCH_3_)]	−2.5	−0.161	0.153	3.264	0.084
[Pt(NH_3_)_2_I(imi)]	−7.6	−0.148	0.160	3.123	0.068
[Pt(NH_3_)I_2_(CH_3_SH)]	3.7	−0.159	0.142	3.533	0.089
[Pt(NH_3_)I_2_(CH_3_S^−^)]	−19.7	−0.123	0.140	3.578	0.054
[Pt(NH_3_)I_2_(CH_3_SCH_3_)]	−0.5	−0.157	0.142	3.516	0.086
[Pt(NH_3_)I_2_(imi)]	−3.1	−0.146	0.149	3.358	0.071

**Table 4:** Calculated Fukui Indices ($f^{+}$ and $f^{-}$) for atomic centers in cisplatin and its monosubstituted analogs. In DMF.

Complex	Fukui Index	Pt	N	N	Cl	Cl	N(imi)	S
[Pt(NH_3_)_2_Cl_2_]	$f^{+}$	−0.410	−0.023	−0.023	−0.171	−0.171	n/a	n/a
	$f^{-}$	−0.655	−0.015	−0.015	−0.104	−0.104	n/a	n/a
[Pt(NH_3_)_2_Cl(CH_3_SH)]	$f^{+}$	−0.358	−0.023	−0.031	−0.174	n/a	n/a	−0.137
	$f^{-}$	−0.452	−0.001	−0.009	−0.292	n/a	n/a	−0.055
[Pt(NH_3_)_2_Cl(CH_3_S^−^)]	$f^{+}$	−0.370	−0.001	−0.008	−0.160	n/a	n/a	−0.210
	$f^{-}$	−0.200	0.018	−0.010	−0.057	n/a	n/a	−0.524
[Pt(NH_3_)_2_Cl(CH_3_SCH_3_)]	$f^{+}$	−0.348	−0.019	−0.035	−0.242	n/a	n/a	−0.111
	$f^{-}$	−0.449	0.000	−0.008	−0.281	n/a	n/a	−0.054
[Pt(NH_3_)_2_Cl(imi)]	$f^{+}$	−0.421	−0.010	−0.040	−0.191	n/a	0.003	n/a
	$f^{-}$	−0.476	0.003	−0.023	−0.299	n/a	0.016	n/a
[Pt(NH_3_)Cl_2_(CH_3_SH)]	$f^{+}$	−0.354	−0.025	n/a	−0.179	−0.193	n/a	−0.111
	$f^{-}$	−0.432	0.002	n/a	−0.279	−0.105	n/a	−0.045
[Pt(NH_3_)Cl_2_(CH_3_S^−^)]	$f^{+}$	−0.333	0.002	n/a	−0.162	−0.124	n/a	−0.212
	$f^{-}$	−0.165	0.016	n/a	−0.053	−0.080	n/a	−0.537
[Pt(NH_3_)Cl_2_(CH_3_SCH_3_)]	$f^{+}$	−0.339	−0.022	n/a	−0.155	−0.157	n/a	−0.116
	$f^{-}$	−0.432	0.003	n/a	−0.262	−0.108	n/a	−0.043
[Pt(NH_3_)Cl_2_(imi)]	$f^{+}$	−0.400	−0.019	n/a	−0.175	−0.169	0.007	n/a
	$f^{-}$	−0.461	0.000	n/a	−0.115	−0.251	0.027	n/a

**Table 5:** Calculated Fukui indices ($f^{+}$ and $f^{-}$) for atomic centers in iodoplatin and its monosubstituted analogs. In DMF.

Complex	Fukui Index	Pt	N	N	I	I	N(imi)	S
[Pt(NH_3_)_2_I_2_]	$f^{+}$	−0.300	−0.022	−0.022	−0.244	−0.244	n/a	n/a
	$f^{-}$	−0.344	0.005	0.005	−0.287	−0.287	n/a	n/a
[Pt(NH_3_)_2_I(CH_3_SH)]	$f^{+}$	−0.303	−0.024	−0.027	−0.280	n/a	n/a	−0.117
	$f^{-}$	−0.220	−0.024	0.003	−0.637	n/a	n/a	−0.026
[Pt(NH_3_)_2_I(CH_3_S^−^)]	$f^{+}$	−0.305	−0.008	−0.007	−0.265	n/a	n/a	−0.191
	$f^{-}$	−0.162	0.022	−0.009	−0.089	n/a	n/a	−0.528
[Pt(NH_3_)_2_I(CH_3_SCH_3_)]	$f^{+}$	−0.307	−0.024	−0.025	−0.275	n/a	n/a	−0.100
	$f^{-}$	−0.220	−0.011	0.004	−0.633	n/a	n/a	−0.024
[Pt(NH_3_)_2_I(imi)]	$f^{+}$	−0.340	−0.016	−0.035	−0.307	n/a	0.004	n/a
	$f^{-}$	−0.242	−0.010	−0.001	−0.640	n/a	0.029	n/a
[Pt(NH_3_)I_2_(CH_3_SH)]	$f^{+}$	−0.248	−0.020	n/a	−0.234	−0.235	n/a	−0.107
	$f^{-}$	−0.205	−0.005	n/a	−0.572	−0.119	n/a	−0.020
[Pt(NH_3_)I_2_(CH_3_S^−^)]	$f^{+}$	−0.253	−0.007	n/a	−0.220	−0.179	n/a	−0.194
	$f^{-}$	−0.157	0.025	n/a	−0.080	−0.119	n/a	−0.484
[Pt(NH_3_)I_2_(CH_3_SCH_3_)]	$f^{+}$	−0.256	−0.018	n/a	−0.230	−0.233	n/a	−0.089
	$f^{-}$	−0.209	−0.004	n/a	−0.562	−0.122	n/a	−0.019
[Pt(NH_3_)I_2_(imi)]	$f^{+}$	−0.284	−0.020	n/a	−0.255	−0.238	0.010	n/a
	$f^{-}$	−0.222	0.002	n/a	−0.250	−0.447	0.017	n/a

## Data Availability

The data underlying this article will be shared on reasonable request to the corresponding authors.

## Supplementary Materials

The supporting information can be downloaded at https://www.mdpi.com/article/10.3390/ijms262412138/s1.

## References

[1] Johnstone T.C., Suntharalingam K., Lippard S.J. (2016). The Next Generation of Platinum Drugs: Targeted Pt(II) Agents, Nanoparticle Delivery, and Pt(IV) Prodrugs. Chem. Rev..

[2] Romani A.M.P. (2022). Cisplatin in Cancer Treatment. Biochem. Pharmacol..

[3] Mariconda A., Ceramella J., Catalano A., Saturnino C., Sinicropi M.S., Longo P. (2025). Cisplatin, the Timeless Molecule. Inorganics.

[4] Oun R., Moussa Y.E., Wheate N.J. (2018). The Side Effects of Platinum-Based Chemotherapy Drugs: A Review for Chemists. Dalton Trans..

[5] Elmorsy E.A., Saber S., Hamad R.S., Abdel-Reheim M.A., El-kott A.F., AlShehri M.A., Morsy K., Salama S.A., Youssef M.E. (2024). Advances in Understanding Cisplatin-Induced Toxicity: Molecular Mechanisms and Protective Strategies. Eur. J. Pharm. Sci..

[6] Fu R., Zhao B., Chen M., Fu X., Zhang Q., Cui Y., Hu X., Zhou W. (2023). Moving beyond Cisplatin Resistance: Mechanisms, Challenges, and Prospects for Overcoming Recurrence in Clinical Cancer Therapy. Med. Oncol..

[7] Sooriyaarachchi M., George G.N., Pickering I.J., Narendran A., Gailer J. (2016). Tuning the Metabolism of the Anticancer Drug Cisplatin with Chemoprotective Agents to Improve Its Safety and Efficacy. Metallomics.

[8] Mitchell E., Pham M.H., Clay A., Sanghvi R., Williams N., Pietsch S., Hsu J.I., Øbro N.F., Jung H., Vedi A. (2025). The Long-Term Effects of Chemotherapy on Normal Blood Cells. Nat. Genet..

[9] Liu B., Zhou H., Tan L., Siu K.T.H., Guan X.-Y. (2024). Exploring Treatment Options in Cancer: Tumor Treatment Strategies. Signal Transduct. Target. Ther..

[10] Casini A., Pöthig A. (2024). Metals in Cancer Research: Beyond Platinum Metallodrugs. ACS Cent. Sci..

[11] Wani M.Y., Malik M.A. (2021). Non-Platinum Anticancer Agents. Gold and Its Complexes in Anticancer Chemotherapy.

[12] Wang Y., Cao B., Wang Q., Zhong S., Fang X., Wang J., Chan A.S.C., Xiong X., Zou T. (2025). Ligand Supplementation Restores the Cancer Therapy Efficacy of the Antirheumatic Drug Auranofin from Serum Inactivation. Nat. Commun..

[13] Su S., Chen Y., Zhang P., Ma R., Zhang W., Liu J., Li T., Niu H., Cao Y., Hu B. (2022). The Role of Platinum(IV)-Based Antitumor Drugs and the Anticancer Immune Response in Medicinal Inorganic Chemistry. A Systematic Review from 2017 to 2022. Eur. J. Med. Chem..

[14] Gibson D. (2021). Pt(IV) Anticancer Prodrugs—A Tale of Mice and Men. ChemMedChem.

[15] Gibson D. (2021). Platinum(IV) Anticancer Agents; Are We En Route to the Holy Grail or to a Dead End?. J. Inorg. Biochem..

[16] Gibson D. (2016). Platinum(IV) Anticancer Prodrugs—Hypotheses and Facts. Dalton Trans..

[17] Chen S., Yao H., Zhou Q., Tse M.-K., Gunawan Y.F., Zhu G. (2020). Stability, Reduction, and Cytotoxicity of Platinum(IV) Anticancer Prodrugs Bearing Carbamate Axial Ligands: Comparison with Their Carboxylate Analogues. Inorg. Chem..

[18] González-Ballesteros M.M., Mejía C., Ruiz-Azuara L. (2022). Metallodrugs: An Approach against Invasion and Metastasis in Cancer Treatment. FEBS Open Bio.

[19] Kim W.K., An J.M., Lim Y.J., Kim K., Kim Y.H., Kim D. (2025). Recent Advances in Metallodrug: Coordination-Induced Synergy between Clinically Approved Drugs and Metal Ions. Mater. Today Adv..

[20] Boros E., Dyson P.J., Gasser G. (2020). Classification of Metal-Based Drugs According to Their Mechanisms of Action. Chem.

[21] Cirri D., Chiaverini L., Pratesi A., Marzo T. (2023). Is the Next Cisplatin Already in Our Laboratory?. Comments Inorg. Chem..

[22] Akitsu T., Tsvetkova D., Yamamoto Y., Nakane D., Kostova I. (2023). From Basics of Coordination Chemistry to Understanding Cisplatin-Analogue Pt Drugs. Curr. Pharm. Des..

[23] Liang W., Huang Y., Wang Y., Lu D., Sun Q. (2025). Research Progress of Platinum-Based Complexes in Lung Cancer Treatment: Mechanisms, Applications, and Challenges. Int. J. Mol. Sci..

[24] Musumeci D., Platella C., Riccardi C., Merlino A., Marzo T., Massai L., Messori L., Montesarchio D. (2016). A First-in-Class and a Fished out Anticancer Platinum Compound: Cis-[PtCl2(NH3)2] and Cis-[PtI2(NH3)2] Compared for Their Reactivity towards DNA Model Systems. Dalton Trans..

[25] Marzo T., Pillozzi S., Hrabina O., Kasparkova J., Brabec V., Arcangeli A., Bartoli G., Severi M., Lunghi A., Totti F. (2015). Cis-Pt I2(NH3)2: A Reappraisal. Dalton Trans..

[26] Quiroga A.G., Cama M., Pajuelo-Lozano N., Álvarez-Valdés A., Perez I.S. (2019). New Findings in the Signaling Pathways of Cis and Trans Platinum Iodido Complexes’ Interaction with DNA of Cancer Cells. ACS Omega.

[27] Tolbatov I., Marzo T., Cirri D., Gabbiani C., Coletti C., Marrone A., Paciotti R., Messori L., Re N. (2020). Reactions of Cisplatin and Cis-[PtI2(NH3)2] with Molecular Models of Relevant Protein Sidechains: A Comparative Analysis. J. Inorg. Biochem..

[28] Pucci R., Angilella G.G.N. (2022). Density Functional Theory, Chemical Reactivity, and the Fukui Functions. Found. Chem..

[29] Wang J., Tao J., Jia S., Wang M., Jiang H., Du Z. (2021). The Protein-Binding Behavior of Platinum Anticancer Drugs in Blood Revealed by Mass Spectrometry. Pharmaceuticals.

[30] Zimmermann T., Chval Z., Burda J.V. (2009). Cisplatin Interaction with Cysteine and Methionine in Aqueous Solution: Computational DFT/PCM Study. J. Phys. Chem. B.

[31] Corinti D., Paciotti R., Coletti C., Re N., Chiavarino B., Crestoni M.E., Fornarini S. (2022). Elusive Intermediates in Cisplatin Reaction with Target Amino Acids: Platinum(II)-Cysteine Complexes Assayed by IR Ion Spectroscopy and DFT Calculations. J. Inorg. Biochem..

[32] Scoditti S., Vigna V., Dabbish E., Sicilia E. (2021). Iodido Equatorial Ligands Influence on the Mechanism of Action of Pt(IV) and Pt(II) Anti-Cancer Complexes: A DFT Computational Study. J. Comput. Chem..

[33] Jansen B.A.J., Brouwer J., Reedijk J. (2002). Glutathione Induces Cellular Resistance against Cationic Dinuclear Platinum Anticancer Drugs. J. Inorg. Biochem..

[34] Messori L., Marzo T., Gabbiani C., Valdes A.A., Quiroga A.G., Merlino A. (2013). Peculiar Features in the Crystal Structure of the Adduct Formed between Cis-PtI_2_ (NH_3_)_2_ and Hen Egg White Lysozyme. Inorg. Chem..

[35] Tolbatov I., Marzo T., Umari P., Mendola D.L., Marrone A. (2025). Detailed Mechanism of a DNA/RNA Nucleobase Substituting Bridging Ligand in Diruthenium(II,III) and Dirhodium(II,II) Tetraacetato Paddlewheel Complexes: Protonation of the Leaving Acetate Is Crucial. Dalton Trans..

[36] Tolbatov I., Umari P., Marrone A. (2024). The Binding of Diruthenium (II,III) and Dirhodium (II,II) Paddlewheel Complexes at DNA/RNA Nucleobases: Computational Evidences of an Appreciable Selectivity toward the AU Base Pairs. J. Mol. Graph. Model..

[37] Tolbatov I., Marrone A. (2022). Reactivity of N-Heterocyclic Carbene Half-Sandwich Ru-, Os-, Rh-, and Ir-Based Complexes with Cysteine and Selenocysteine: A Computational Study. Inorg. Chem..

[38] Scoditti S., Dabbish E., Russo N., Mazzone G., Sicilia E. (2021). Anticancer Activity, DNA Binding, and Photodynamic Properties of a N∧C∧N-Coordinated Pt(II) Complex. Inorg. Chem..

[39] Tolbatov I., Cirri D., Tarchi M., Marzo T., Coletti C., Marrone A., Messori L., Re N., Massai L. (2022). Reactions of Arsenoplatin-1 with Protein Targets: A Combined Experimental and Theoretical Study. Inorg. Chem..

[40] Dunning T.H. (2012). Gaussian Basis Sets Tor Molecular Calculations.

[41] Chai J.-D., Head-Gordon M. (2008). Long-Range Corrected Hybrid Density Functionals with Damped Atom–Atom Dispersion Corrections. Phys. Chem. Chem. Phys..

[42] Remya K., Suresh C.H. (2013). Which Density Functional Is Close to CCSD Accuracy to Describe Geometry and Interaction Energy of Small Noncovalent Dimers? A Benchmark Study Using Gaussian09. J. Comput. Chem..

[43] Andrae D., Häußermann U., Dolg M., Stoll H., Preuß H. (1990). Energy-Adjustedab Initio Pseudopotentials for the Second and Third Row Transition Elements. Theor. Chim. Acta.

[44] Weigend F., Ahlrichs R. (2005). Balanced Basis Sets of Split Valence, Triple Zeta Valence and Quadruple Zeta Valence Quality for H to Rn: Design and Assessment of Accuracy. Phys. Chem. Chem. Phys..

[45] Barone V., Cossi M., Tomasi J. (1997). A New Definition of Cavities for the Computation of Solvation Free Energies by the Polarizable Continuum Model. J. Chem. Phys..

[46] Klamt A., Moya C., Palomar J. (2015). A Comprehensive Comparison of the IEFPCM and SS(V)PE Continuum Solvation Methods with the COSMO Approach. J. Chem. Theory Comput..

[47] Kelly C.P., Cramer C.J., Truhlar D.G. (2007). Single-Ion Solvation Free Energies and the Normal Hydrogen Electrode Potential in Methanol, Acetonitrile, and Dimethyl Sulfoxide. J. Phys. Chem. B.

[48] Isaev Y.I., Makarov D.M., Khodov I.A. (2025). Machine Learning Prediction of NMR Shifts for Rare and Transition Metal Complexes (45Sc, 49Ti, 89Y, 91Zr, 139La). J. Mol. Liq..

[49] Geerlings P., De Proft F., Langenaeker W. (2003). Conceptual Density Functional Theory. Chem. Rev..

[50] Chakraborty D., Chattaraj P.K. (2021). Conceptual Density Functional Theory Based Electronic Structure Principles. Chem. Sci..

[51] Yang W., Mortier W.J. (1986). The Use of Global and Local Molecular Parameters for the Analysis of the Gas-Phase Basicity of Amines. J. Am. Chem. Soc..

