# Supplementary material for: Mechanistic Fingerprints from Chloride to Iodide: Halide vs. Ammonia Release in Platinum Anticancer Complexes
Source: Int J Mol Sci. 2025 Dec 17;26(24):12138. doi: 10.3390/ijms262412138 (PMC12733153; doi:10.3390/ijms262412138)
Supplement: Supplementary file 1 [file ijms-26-12138-s001.zip › ijms-4014876-supplementary.pdf]

Mechanistic Fingerprints from Chloride to Iodide: Halide vs Ammonia Release in Platinum Anticancer Complexes

Contents

^{14}NNMR stability spectra.. 1

Computational details…….4

^{14}NNMR stability spectra

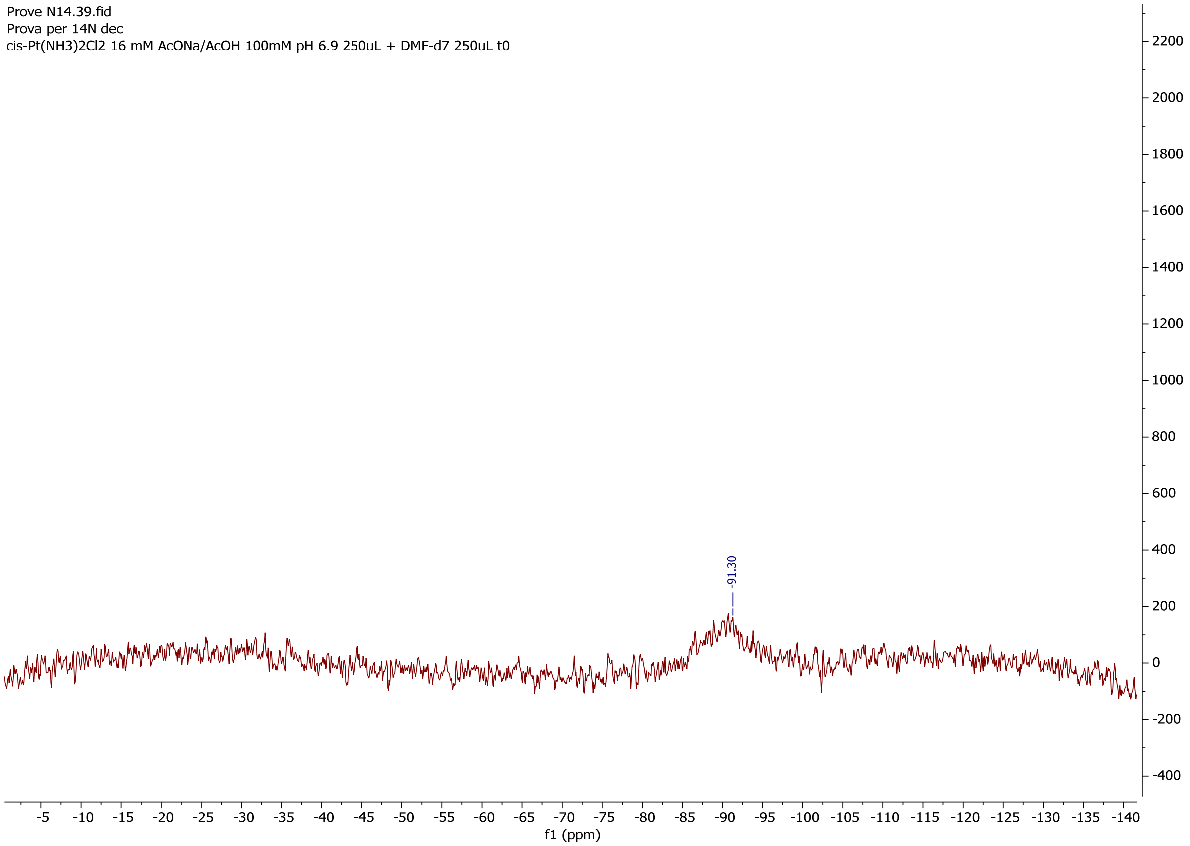

Fig.S1: ^{14}NNMR stability spectrum of cisplatin at t0 in a solvent mixture of 250 µl of DMF-d_7 and 250 µl of Buffer Acetate 100 mM pH 6.9.

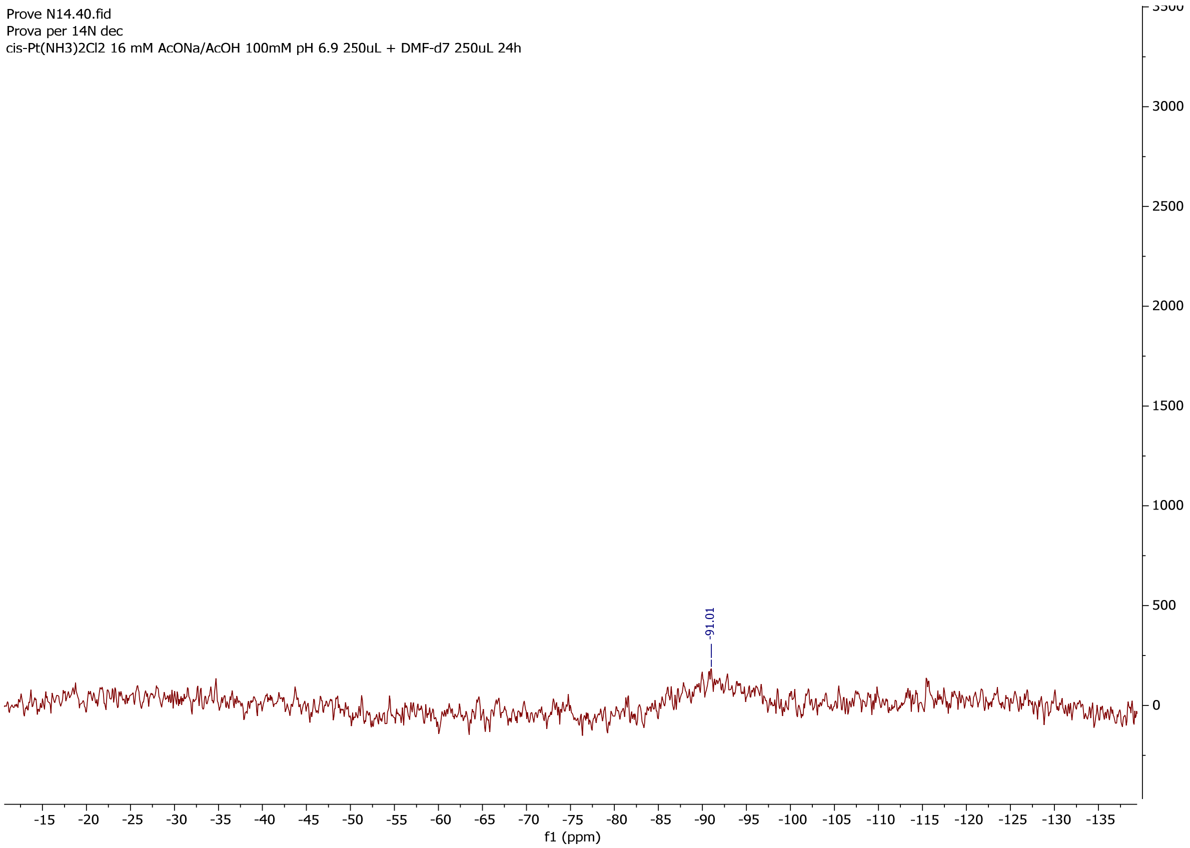

Fig.S2: ^{14}NNMR stability spectrum of cisplatin after 24h in a solvent mixture of 250 µl of DMF-d_7 and 250 µl of Buffer Acetate 100 mM pH 6.9.

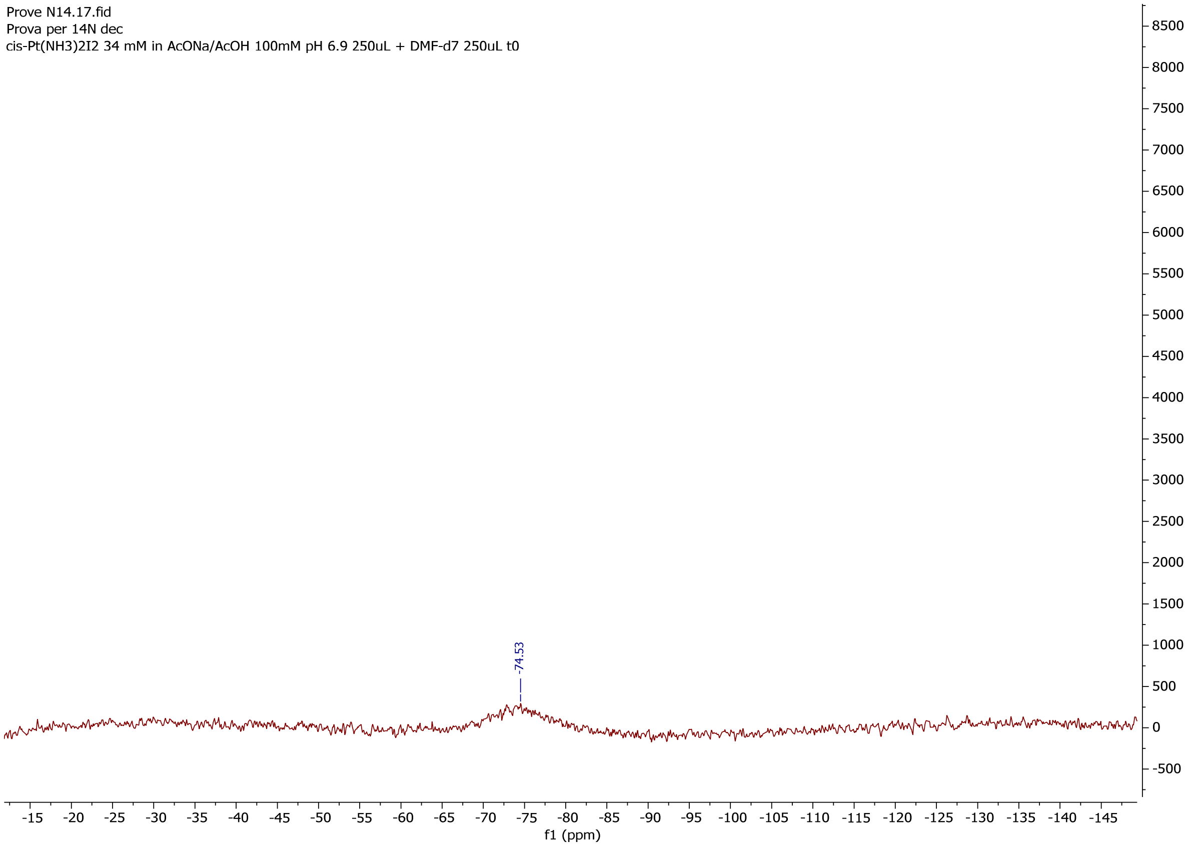

Fig.S3: ^{14}NNMR stability spectrum of cis-[$PtI_2(NH_3)_2$] at t0 in a solvent mixture of 250 µl of DMF-d_7 and 250 µl of Buffer Acetate 100 mM pH 6.9.

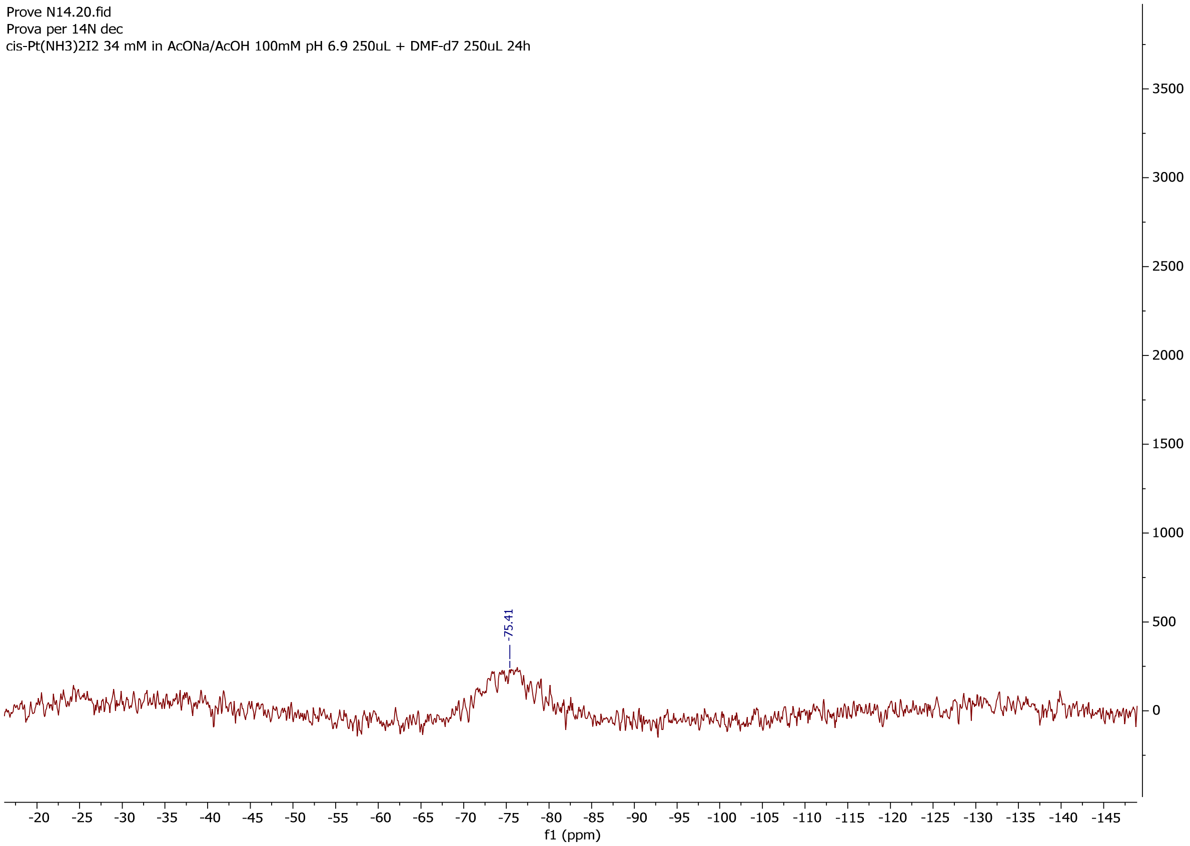

Fig.S4: ^{14}NNMR stability spectrum of cis-[$PtI_2(NH_3)_2$] after 24h in a solvent mixture of 250 µl of DMF-d_7 and 250 µl of Buffer Acetate 100 mM pH 6.9.

Table S1. Gibbs free energies of substitution (GFE in kcal/mol) and conceptual DFT global descriptors (μ,η,S,ω) for cisplatin and monosubstituted analogues. In water.

Complex	GFE	μ	η	S	ω
[$Pt(NH_3)_2Cl_2$]	0	-0.140	0.169	2.953	0.058
[$Pt(NH_3)_2Cl(CH_3SH)$]	4.9	-0.161	0.169	2.966	0.077
[$Pt(NH_3)_2Cl(CH_3SCH_3)$]	0.2	-0.160	0.170	2.942	0.075
[$Pt(NH_3)_2Cl$(imi)]	-4.2	-0.144	0.177	2.819	0.059
[$Pt(NH_3)Cl_2(CH_3SH)$]	5.1	-0.154	0.163	3.071	0.072
[$Pt(NH_3)Cl_2(CH_3SCH_3)$]	1.0	-0.153	0.164	3.053	0.071
[$Pt(NH_3)Cl_2$(imi)]	-1.6	-0.138	0.169	2.954	0.056

Table S2. Gibbs free energies of substitution (GFE in kcal/mol) and conceptual DFT global descriptors (μ, η, S, ω) for cisPtI_2 and monosubstituted analogues. In water.

Complex	GFE	*μ*	*η*	*S*	*ω*
$[Pt(NH_3)_2I_2]$	0	-0.149	0.148	3.386	0.075
$[Pt(NH_3)_2I(CH_3SH)]$	2.2	-0.161	0.152	3.279	0.085
$[Pt(NH_3)_2I(CH_3SCH_3)]$	-2.3	-0.159	0.153	3.259	0.082
$[Pt(NH_3)_2I(imi)]$	-7.4	-0.146	0.160	3.117	0.067
$[Pt(NH_3)I_2(CH_3SH)]$	4.0	-0.159	0.142	3.528	0.089
$[Pt(NH_3)I_2(CH_3SCH_3)]$	-0.1	-0.157	0.142	3.511	0.087
$[Pt(NH_3)I_2(imi)]$	-2.9	-0.146	0.149	3.352	0.071

Table S3. Calculated Fukui Indices (f^+ and f^-) for atomic centers in cisplatin and its monosubstituted analogues. In water.

complex	Fukui index	Pt	N	N	Cl	Cl	N(imi)	S
$[Pt(NH_3)_2Cl_2]$	f^+	-0.414	-0.024	-0.024	-0.169	-0.169	n/a	n/a
	f^-	-0.517	-0.001	-0.001	-0.172	-0.172	n/a	n/a
$[Pt(NH_3)_2Cl(CH_3SH)]$	f^+	-0.361	-0.024	-0.031	-0.172	n/a	n/a	-0.136
	f^-	-0.462	-0.001	-0.009	-0.286	n/a	n/a	-0.054
$[Pt(NH_3)_2Cl(CH_3SCH_3)]$	f^+	-0.362	-0.022	-0.030	-0.166	n/a	n/a	-0.121
	f^-	-0.459	0.000	-0.009	-0.274	n/a	n/a	-0.053
$[Pt(NH_3)_2Cl(imi)]$	f^+	-0.424	-0.011	-0.041	-0.189	n/a	0.002	n/a
	f^-	-0.485	0.004	-0.024	-0.292	n/a	0.016	n/a
$[Pt(NH_3)Cl_2(CH_3SH)]$	f^+	-0.340	-0.024	n/a	-0.161	-0.158	n/a	-0.134
	f^-	-0.442	0.002	n/a	-0.275	-0.100	n/a	-0.044
$[Pt(NH_3)Cl_2(CH_3SCH_3)]$	f^+	-0.344	-0.023	n/a	-0.154	-0.155	n/a	-0.116
	f^-	-0.443	-0.003	n/a	-0.258	-0.104	n/a	-0.043
$[Pt(NH_3)Cl_2(imi)]$	f^+	-0.404	-0.020	n/a	-0.173	-0.168	0.006	n/a
	f^-	-0.470	0.000	n/a	-0.112	-0.247	0.026	n/a

Table S4. Calculated Fukui indices (f^+ and f^-) for atomic centers in iodoplatin and its monosubstituted analogues. In water.

complex	Fukui index	Pt	N	N	I	I	N(imi)	S
[Pt(NH$_3$)$_2$I$_2$]	f^+	-0.302	-0.022	-0.022	-0.245	-0.245	n/a	n/a
	f^-	-0.359	0.004	0.004	-0.278	-0.278	n/a	n/a
[Pt(NH$_3$)$_2$I(CH$_3$SH)]	f^+	-0.308	-0.025	-0.028	-0.278	n/a	n/a	-0.116
	f^-	-0.226	-0.011	0.004	-0.633	n/a	n/a	-0.025
[Pt(NH$_3$)$_2$I(CH$_3$SCH$_3$)]	f^+	-0.312	-0.025	-0.026	-0.273	n/a	n/a	-0.100
	f^-	-0.226	-0.010	0.004	-0.630	n/a	n/a	-0.023
[Pt(NH$_3$)$_2$I(imi)]	f^+	-0.345	-0.017	-0.035	-0.304	n/a	0.004	n/a
	f^-	-0.249	-0.009	-0.002	-0.635	n/a	0.029	n/a
[Pt(NH$_3$)I$_2$(CH$_3$SH)]	f^+	-0.254	-0.021	n/a	-0.232	-0.232	n/a	-0.107
	f^-	-0.212	-0.005	n/a	-0.571	-0.114	n/a	-0.019
[Pt(NH$_3$)I$_2$(CH$_3$SCH$_3$)]	f^+	-0.261	-0.019	n/a	-0.228	-0.230	n/a	-0.089
	f^-	-0.216	-0.004	n/a	-0.562	-0.118	n/a	-0.018
[Pt(NH$_3$)I$_2$(imi)]	f^+	-0.289	-0.021	n/a	-0.252	-0.236	0.009	n/a
	f^-	-0.097	0.000	n/a	-0.354	-0.482	0.014	n/a
